# The envy-contempt spiral: affective self-regulation in grandiose narcissism

**DOI:** 10.3389/fpsyt.2025.1620201

**Published:** 2025-06-24

**Authors:** Alexandros Raftopoulos, Human-Friedrich Unterrainer

**Affiliations:** ^1^ Faculty of Psychotherapy Science, Sigmund Freud University, Vienna, Austria; ^2^ Department of Religious Studies, University of Vienna, Vienna, Austria; ^3^ University Clinic for Psychiatry and Psychotherapeutic Medicine, Medical University Graz, Graz, Austria

**Keywords:** affective neuroscience, contempt, envy, grandiose narcissism, psychodynamic theory, self-regulation

## Abstract

In classic psychoanalytic theory, narcissism and envy have been theorized to be inseparably interwoven. Nevertheless, empirical findings have not yet been able to substantiate this relationship. Conversely, most studies showed that grandiose facets of narcissism curbed feelings of envy, suggesting an envy-protection inherent to grandiose narcissism. Consistent with these findings, contemporary psychodynamic accounts, specifically object-relations theory, conceptualize grandiose narcissism as a defensive structure against envy via the elicitation of contempt. In the present paper, we translate this theory to contemporary personality psychology by drawing on Affective Neuroscience and socio-functional approaches of emotion. We propose that envy and contempt interact in a self-regulating, opposing way, forming the core of the self-protective strategy seen in grandiose narcissism. Placing this self-regulatory emotional dynamic at the center of grandiose narcissism, we present an affect-centric process model that aims to explain antagonistic self-protective behaviors shown by individuals high on grandiose narcissism. Specifically, we conceptualize these self-protective processes as rooted in a strong status motive, combined with a stable tendency to experience envy in response to upward comparisons that pose ego or status threats. To regulate envy, we propose that contempt is automatically activated, leading to devaluation through indifference, which in turn fosters social conflict. We further apply these dynamics to explain the change of relationship trajectories (short-term acquaintance vs. long-term acquaintance) of individuals high on grandiose narcissism, by suggesting the envy-contempt dynamic to exacerbate in long-term acquaintances, in which individuals high on grandiose narcissism tend to defend against the uprise of feelings of dependency on their partners admiration. While supporting empirical findings are outlined throughout the article, we finally propose a variety of questions that should be addressed in the future in order to scrutinize our model.

## 1 Introduction

The concept of narcissism has been subject to an exponential explosion in scientific attention in the past few decades (1), resulting in a proliferation of theories, measures, empirical findings and clinical classifications. However, due to its elusive and paradoxical phenotype (2), as well as the panorama of narcissistic phenomena beyond definitions of self-centeredness and egotism (3), a common consensus or a shared framework about what ultimately constitutes narcissism has not yet been found. In light of the history of the concept, this comes as no surprise, as it has been dividing the minds of clinicians and researchers all along – initially in psychoanalytic theory –, having been a crossroad in Freud’s drive theory (4), but also more recently sparking a debate between the two clinical narcissism experts Heinz Kohut and Otto F. Kernberg, which centers around the question whether pathological narcissism stems from the fixation and regression on a normal early childhood phase (5) or is constituted by a pathological compensatory structure, that categorically rather than dimensionally differs from normal childhood narcissism (6, 7). This mainly clinical debate has carried over to social and personality psychology, where it fueled discussions about (a) the key features of narcissism, (b) how these features are organized and related to each other and (c) why they are organized that way (1). Consistent with a broader shift in personality psychology towards a more process-oriented conceptualization of personality, numerous process models have been developed in order to address these questions (e.g. 2, 8, 9). These models commonly posit that the narcissist’s overarching goal is the maintenance of both their grandiose self and status, which is achieved by either a self-enhancing or a self-protective strategy. Although, however, most of these models incorporate an affective or emotional facet of narcissistic dynamics, we argue that relatively little emphasis has been placed on identifying the specific affects or emotions that constitute narcissism. To bridge this gap, we propose a neuroscientifically informed self-regulatory process model that highlights two distinct affects that are hypothesized to be in an antagonistic relation to each other and are thought to constitute the motor of narcissistic self-protective dynamics. Similar to the *shame-rage* sp*iral* (10) that has been linked to vulnerable narcissism (11), we propose an envy-contempt spiral that aims to explain grandiose-narcissistic, more precisely antagonistic self-protective behaviors. In doing so, we also translate classic psychoanalytic theory, namely object relations theory (6, 7, 12), into contemporary personality psychology.

In this study, we will provide a succinct outline of relevant advances in social- and personality psychology that inform our proposed model. This will include the recent shift towards process-oriented conceptualizations of personality, as well as the increasingly recognized importance of affects and emotions as constituents of personality (13). We will then elaborate on process models of grandiose narcissism that serve as the foundation of our model. This will lead us to narcissistic emotions, the role of envy and emotion regulation characteristic for grandiose narcissism. Finally, we will introduce contempt as a defensive emotion exhibited by grandiose narcissists. Having outlined the presuppositions of our model, we will then densify its components in order to construct the complete model and conclude the article with suggestions for future validations of the underlying hypotheses.

## 2 Personality dynamics, emotions and their regulation

Whereas traditional personality theories emphasize stable interindividual differences in personality traits, more recent approaches have shifted toward models that also consider meaningful intraindividual variability, thereby capturing personality dynamics (14, 15). In response to this shift, several theoretical frameworks have emerged, including the cognitive-affective processing system (CAPS; 16), trait activation theory (TAT; 17), and whole trait theory (WTT; 18). Building on these models, multiple personality traits (including grandiose narcissism; 19) have been studied by examining how stable dispositions (the nonconditional aspects) interact with situational, context-dependent factors (the state components). Apart from research on personality dynamics, advances in the field of affects have profoundly influenced personality psychology. Although classic personality theories have long conceived of affects as subsystems of the mind, an in-depth examination has only fairly recently been made possible through the reconciliation of personality and emotion psychology (20). Moreover, psychodynamic studies have experienced a resurgence with the help of Affective Neuroscience (21–23) and have been integrated to contemporary conceptualizations of emotions (24, 25). However, a central point of disagreement between these theories remains in regards to the question how the relationship between cognition and affect is to be conceptualized. Classic personality and emotion theories suggest a primacy of cognition over affect, the latter therefore only secondarily being elicited through a primary cognitive appraisal of a situation (26). Conversely, Affective Neuroscience turns this hierarchy upside down, by postulating both a temporal and a neurobiological primacy of affect over cognition (23, 27, 28). According to this line of research, primary processes, conceptualized as primary emotional systems and viewed analogously to instincts, function independently of any intervening cognitive reflections. Moreover, Panksepp (21) proposes seven primary systems shared by all mammals, including humans. Primary-process systems encompass the following: SEEKING/foraging, PLAY/joy, CARE/nurturance, LUST/sexuality; PANIC/separation, RAGE/anger, and FEAR/anxiety (29–31). These systems not only support subjective affective experiences that motivate and guide our actions, control learning and memory, and promote diverse cognitive activities, they may also, and this is where Affective Neuroscience goes a step further than classic personality psychology and emotion psychology, constitute personality in its core (13, 31).

These advances have also contributed to the field of emotion regulation, which has largely been dominated by cognitive tradition, with the most established model being Gross’ (26, 32) Cognitive Emotion Regulation model. In line with the Appraisal theory of emotion, this model posits that emotion regulation can be achieved and promoted by using regulatory strategies on a behavioral, cognitive or attentional level (26, 27). In contrast to these models, we pay tribute to neuroscientifically informed psychodynamic frameworks that focus on dysregulated affective states due to defensive affects (33). By intertwining classic psychoanalytic theory of defense mechanisms with Affective Neuroscience, the Experiential-Dynamic Emotion Regulation framework (EDER; 34–36) aims to add new facets to the understanding of emotional (dys-)regulation. The EDER holds that emotional responses are prewired at birth with inborn adaptive action tendencies and facial expressions which precede cognition (36). Furthermore, in line with neuroscientific research, emotions are conceptualized as implicitly generated by subcortical brain structures. Essentially, according to the EDER, *defensive affects* play a central role in affective dysregulations, as they are understood to be mobilized excessively and maladaptively (33, 37–40). Over the course of this article we will show how we adopt parts of this framework to conceptualize a self-regulatory dynamic that, we propose, is inherent to narcissistic self-protection.

Our model will couple these parallelly running advances by hypothesizing (a) personality to be constituted by stable process dynamics and (b) the very core of these processes to be constituted by specific affects and their regulatory interplay. Leaning on contemporary approaches that conceive of personality as stable self-regulatory mechanisms (41–43), we go a step further and conceptualize emotional dynamics to be the building blocks of personality traits. More precisely, our proposed model can therefore be understood as informed by a combination of the above mentioned models. We borrow the general idea of *cognitive-affective units*, as well as of *if-then* contingencies from the CAPS and also integrate regulatory processes described in Gross’ model. The model is also informed by the central aspects of the WTT, as we suppose narcissistic strategies to fluctuate meaningfully intraindividually across situations. The TAT, on the other hand, complements these aspects by emphasizing the role of trait-relevant situational cues, as will be shown below. However, in line with the EDER and the neuroscientific research that it is built upon, we posit a primacy of emotion over cognition and especially in terms of regulatory processes emphasize the importance of defensive affects.

Terminologically and taxonomically, emotions and affects are often used synonymously, while others use them more distinctly. Gross (26), for example, uses affects as a superordinate category for valenced states, under which he subsumes distinct emotions, as well as emotional episodes, moods and dispositional emotions. In Affective Neuroscience, affects are understood to be subjectively experienced aspects of emotions (i.e., feelings). Occasionally, the terms are used in combination, coining the term emotional affects, which are connected to primary-process systems (44). We will be using the term “emotion” and respectively “emotion regulation” to refer to subjective experiences and their regulation and will differentiate between primary or basic and secondary emotions whenever necessary.

## 3 Grandiose narcissism

Because of its multifaceted nature, narcissism represents both a highly intriguing and challenging area of research (1). Its complex and paradoxical features (2) have made it difficult to integrate into a single, unified framework. While it was initially conceptualized as a unitary construct, there is growing consensus that it comprises multiple dimensions (45). However, disagreement about the underlying factor structure still remains: while few studies found narcissism to consist of five factors (46, 47), the larger body of evidence points to three (48–50) or two factor solutions (50–52). More recent studies build on the latter, partitioning narcissism into grandiose and vulnerable dimensions. Grandiose narcissism is associated with a sense of superiority, entitlement, extraversion, high self-esteem and higher life satisfaction (1, 51, 53–56), as well as greater sensitivity to ego-threats (57). Individuals high on grandiose narcissism further tend to thrive in new social context, as they appear particularly charming and admirable at short-term acquaintance (58–60). Paradoxically, grandiose narcissism is also linked to dominant and hostile behaviors and less popularity in long-term acquaintance contexts (2, 61–64). Vulnerable Narcissism, on the other hand, is linked to low self-esteem, introversion (51, 65–68) propensity for shame and envy, as well as avoidant reactions to others (69).

As noted above, the past decade has seen a shift toward conceptualizing personality as interindividual differences in intraindividual variability. These approaches focus on processes that explain personality dynamics (e.g., 18, 70). This perspective has also been applied to narcissism, particularly to grandiose narcissism, which is often seen as particularly paradoxical. Due to its conflicting cognitive, affective-motivational, behavioral, and social features, complex process models have been developed to capture its underlying dynamics. Among these, the Narcissistic Admiration and Rivalry Concept (NARC; 8) is one of the most prominent.The NARC proposes two separate self-regulatory strategies, that are positively related but have different nomological networks, through which the narcissists overarching goal, the maintenance of grandiose self, is achieved: (a) an assertive self-enhancement strategy, termed narcissistic admiration and (b) an antagonistic self-protection strategy, termed narcissistic rivalry. Narcissistic admiration sets in motion a process characterized by striving for uniqueness, grandiose fantasies and charmingness, that leads to social potency, which ultimately feeds back into and reinstates this dynamic. On the contrary, narcissistic rivalry initiates a striving for supremacy, devaluation of others and aggressiveness - a set of behavioral dynamics that give rise to rejection, unpopularity and social conflict overall.

In their Process Model of Narcissistic Status Pursuit (SPIN), Grapsas et al. (9), combine these processes of grandiose narcissism with the above-mentioned process models of emotion regulation (26), to zoom in on moment-by-moment dynamics. They apply core self-regulatory processes (i.e., situational selection, vigilance, appraisal and response execution) to explain the why and how of grandiose narcissism. More precisely, the process of status pursuit unfolds as follows: firstly, grandiose narcissists tend to select situations in which they believe status can be increased. Secondly, once such situations are encountered, grandiose narcissists become particularly vigilant to whether the situation facilitates or hinders status pursuit. Thirdly, respective of whether status pursuit is facilitated or hindered, they appraise either self-enhancing or antagonistic self-protective as purposeful for status maintenance (9).

The aim of this article is to further zoom in on these dynamics by both, borrowing elements of existing models as well as adding new aspects, and to ultimately develop a new model that focuses on emotional dynamics of grandiose narcissism with a main focus on antagonistic self-protection. Occasionally, we will be referring to vulnerable narcissism as well as narcissistic admiration and will specify this accordingly. We will also be referring to other process models, which will, however, only be elaborated to the extent that is relevant to the presented argumentation.

## 4 Narcissistic emotions

In psychoanalytic literature and practice, narcissism was initially linked to envy by Freud (71). In the later developments of psychoanalytic theory, especially in Kleinian tradition, envy was somewhat decoupled from narcissism, even though it continued to relate to it implicitly. Kernberg (7), who is known for his work on narcissistic personality, explicated the relation between envy and narcissism to lay the groundwork for his narcissism theory. According to Kernberg, narcissists “experience a remarkably intense envy of other people who seem to have things they do not have or who simply seem to enjoy their lives”. Kohut (5), on the other hand, emphasizes not envy, but shame as inherent to narcissism. According to his etiopathogenetic model, narcissism is constituted by the construction of a grandiose-self that helps to compensate the loss of primary narcissism (p. 43). While in early childhood this configuration is deemed as adaptive, over the course of the early childhood it has to be overcome by means of what Kohut calls *optimal frustration*. Under favorable circumstances, the result is what Kohut calls a *transmuting internalization*, which refers to the building of intrapsychic structures. However, in case the relationship to primary caregivers fails to optimally frustrate the child’s notion of grandiosity, this internalization is rendered impossible, resulting in a fixation in and a later regression to this narcissistic stage. This chronic regression, that culminates in pathological narcissism, is, according to Kohut, characterized by infantile grandiosity, self-centeredness and more importantly shame propensity (5, 69).

Psychoanalytic theories have often resisted empirical testing because they focus on unconscious processes, which are only indirectly observable through secondary expressions (72). Accordingly, these theories - however fruitful they might seem - have a history of being underrepresented in empirical science and only recently have started being conceptualized and operationalized in ways that allow them to be tested empirically. In terms of narcissism this recent change is particularly reflected in the development of process models that respect the psychoanalytic roots of the concept (see above). Specifically, recent research on the emotional lives of narcissists has been inspired by psychodynamic theory. Nevertheless, prior research has mostly focused on anger and shame in line with Kohutian theory, whereas emotions theorized in object-relations, especially envy, have been left somewhat aside (69).

There is now increasing agreement that narcissism is associated with a tendency toward shame, often referred to as the shame-rage spiral (10, 73, 74) or narcissistic rage (11). Narcissistic rage is defined as a vitriolic, retaliatory response to a perceived status threat (75).A large body of evidence reveals that narcissists react aggressively when they are confronted with strong threats to self (76–83), with the goal of imposing superiority, through which they strive to restore the threatened self (11, 76). Narcissistic rage, however, should not be reduced to provoked aggression, as it consists of three core features, (a) anger and hostility, (b) shame and inferiority and (c) displaced aggression (for details, see 11). The narcissistic rage hypothesis has recently been examined empirically by Krizan & Johar (11), who were able to find strong support for the link between vulnerable narcissism and narcissistic rage. Not unexpectedly, grandiosity seemed not to be correlated with narcissistic rage.

Another example for the entry of Kohutian theory into personality psychology is the model by Tracy et al. (84), who draw on early clinical and contemporary accounts to develop an emotion-centered model that emphasizes shame and pride as the driving forces of narcissism. According to the authors, these emotions result from a split in the self-representational system and subsequently a split in self-esteem. This leads to a coexistence of feelings of grandiosity and hubristic pride on one side, and intense feelings of inadequacy and shame on the other side – the latter being kept implicit by means of a defensive self-regulatory style of compensatory nature (85).

Other models that have contributed to the understanding of the emotional lives of narcissists have shifted the focus away from identifying single emotions. As outlined above, the NARC (8) conceptualizes grandiose narcissism as motivated by the maintenance of grandiose-self, which can be achieved by two social strategies, that both encompass an affective-motivational domain. However, as the authors self-critically remark, emotions have not been examined specifically, let alone self-conscious emotions. The same applies to Grapsas et al. (9) process model that combines the NARC with Gross’ emotion regulation model, while paradoxically does not consider emotions or affects at all. In a separate study the same group of researchers investigated the affective contingencies of narcissism and even applies physiological methods to do so (86). Even though their findings suggest that narcissists tend to react positively to power satisfactions, their overall findings do not substantiate a robust relation between affective contingencies and narcissists’ behavior.

In a recent publication, Kroencke et al. (87) developed and empirically validated a process approach that is based on the three-factor models of narcissism (i.e. accounts for antagonistic, agentic and neurotic aspects) and incorporates a more differentiated view on emotions. This model is one of the very few process models that link antagonistic narcissism to envy. Specifically, the authors associate (a) agentic narcissism with positive emotions, such as pride and joy, whenever a status gain is achieved, (b) neurotic narcissism with shame and (c) antagonistic narcissism with envy and anger.

## 5 Envy and narcissism

As already indicated above, narcissism and envy are historically inextricably interwoven. The first conceptual bridge between the two phenomena was built by Freud (71) who suggested that envy was both the result of as well as the driving force to overcome a narcissistic wound. Later, Melanie Klein, who defined envy as “the angry feeling that another person possesses and enjoys something desirable - the envious impulse being to take it away or to spoil it” (12, p. 290), elaborated the envy concept and prepared a fertile ground for Kernberg to reconnect it to narcissism. Moreover, envy is listed as one of the diagnostic criteria in the Diagnostic and Statistical Manual of Mental Disorders (DSM-IV; 88).

In social- and personality psychology, envy enjoys a growing body of evidence. It is understood as a complex and multifaceted emotion (89), defined as an unpleasant, often painful emotion characterized by feelings of inferiority, hostility, and resentment produced by an awareness of another person or group of persons who enjoy a desired possession (object, social position, attribute, or quality of being) (90). Envy always originates from an upward comparison (91) followed by the motivation to equalize the perceived difference between envier and envied (92). In line with this, envy has been understood to be an emotional reaction to status pursuit in social hierarchies (91, 93). Moreover, envy has been conceptualized as a comparison-based emotional trait, so-called dispositional envy, to account for interindividual differences in stable tendencies to both seek out upward comparisons as well as feel envy in case of comparisons with undesirable outcomes (94, 95). Dispositional envy has further been conceptualized as both a dual construct, consisting of a benign and a malicious dimension (92, 96), as well as a domain-specific trait, encompassing three domains: attraction, competence and wealth (95).

Ultimately, narcissism aims to preserve grandiosity and status. Since envy arises from upward comparisons related to status, the connection between the two is evident. Nevertheless, supporting evidence for the relation between narcissism and envy remained inexistent for a long time and only started being systematically investigated in the last decade. In the first systematic examination (69), vulnerable narcissism was strongly linked to dispositional envy, while grandiose narcissism proved to be slightly negatively linked to dispositional envy, hinting at a built-in envy-protection potentially characteristic for grandiose narcissism. Neufeld and Johnson (97) expanded on the relation between narcissism and envy and confirmed the previous findings, suggesting that the link between grandiose narcissism and envy is more complex. In line with previous studies, the authors also confirmed that grandiose narcissism seemed to curb envy feelings. However, Lange et al. (93), contrasted these findings by examining both, narcissism and envy, in regards to their multidimensionality. Building on the NARC, they found robust links between (a) narcissistic admiration and benign envy via hope for success and (b) narcissistic rivalry with malicious envy via fear of failure.

## 6 Narcissism and emotion regulation

Given that the goal of individuals high on grandiose narcissism is the maintenance of their grandiose-self or social status, the threat or impeachment of the same sets narcissistic dynamics in motion and subsequently gives rise to strong affective reactions (87, 98). These reactions have to be down-regulated in order for social functioning to remain intact and mental health to be sustained, as difficulties in emotion regulation have been identified as a risk factor for mental health (34, 99–103). As already alluded to above, while vulnerable narcissism has been consistently associated with emotional instability and deficits in emotion regulation (11, 104), the evidence regarding grandiose narcissism remains more ambiguous (105, 106). Some studies even indicate a negative association with emotion dysregulation, suggesting that individuals high on grandiose narcissism could maintain confidence and tolerate setbacks in pursuit of their goals (107).

Furthermore, as mentioned above, grandiose narcissism is understood as a defensive structure against threats and impeachments to social status and feelings of inferiority more generally (7). Originally, feelings of inferiority, insecurity and low self-esteem were attributed to the vulnerable dimension of narcissism, which is why grandiosity was thought to defend against vulnerability. However, empirical studies have shown that, in contrast to this conceptualization, vulnerable and grandiose dimensions of narcissism constitute distinct, rather than negatively related factors in the sense of a defensive interplay (108, 109). Nevertheless, grandiose narcissism did not cease to be examined in regards to its defensive structure and accordingly has been found to be linked to a plethora of defense mechanisms. Associations between narcissism and devaluation, omnipotence, idealization and mood-incongruent denial (110, 111) and grandiose fantasies (112) have been found. Findings regarding the so-called repression hypothesis further confirm that grandiosity is associated with an initial activation and a subsequent unconscious inhibition of feelings of worthlessness when confronted with ego-threats (113, 114). Narcissists also tend to devalue the source of the ego-threat (78, 79, 115). Moreover, a recent study has disentangled the relation between narcissism and defense mechanisms by systematically linking grandiosity and vulnerability to specific defense mechanisms, while also analyzing indirect effects on psychological distress (52). Although findings showed that grandiose narcissism had no direct effects on psychological distress (in contrast to vulnerable narcissism, which was strongly linked to distress), it showed significant associations when mediated by maladaptive defense mechanisms, such as devaluation.

Taken together, the evidence on emotion regulation and defense mechanisms characteristic for grandiose narcissism remains elusive, but is opening up possibilities for theoretical considerations at the same time. Given the fact that grandiose narcissism can be understood as a defensive or self-protective structure, we ask what it is that grandiose narcissism defends and protects against exactly and how this is achieved? While the answer to this may seem trivial, especially in light of the above outlined models (i.e., grandiose narcissism antagonistically self-protects against status threats), we posit that zooming in on the exact emotions that are in play offers a novel approach in understanding the emotional lives of individuals high on grandiose narcissism more deeply. Accordingly, in line with clinical accounts and the above outlined findings in regards to grandiose narcissism as an envy-protective trait, we hypothesize that grandiose narcissism’s main objective is to defend against feelings of envy.

## 7 Contempt – envy’s antidote

Coming back to psychoanalytic theory, we build on Klein (12) and Kernberg (7) in order to propose an affective interplay between two emotions that, as we hypothesize, is constitutive for narcissistic behavior. In her pioneering work *on envy and gratuity*, Klein not only emphasized the importance of envy itself but also the defense mechanisms that are mobilized to counteract and keep envy unconscious, a central one being the act or process of devaluation. Kernberg expanded this idea by connecting it to grandiose narcissism, emphasizing contempt as the emotion driving devaluation and derogation:: By means of conscious or unconscious devaluation, these patients try to defend themselves against emerging feelings of envy. On a conscious level, this manifests itself in a lack of interest in other people and their work or activities as well as a feeling of contempt, which can vary in intensity.” (7, p. 265).

But what exactly is contempt and which function does it serve? While this has always been subject of debate in philosophy, relevant empirical research has only started to answer these questions in the past two decades (116), and only very recently on the level of personality (117). All along, contempt enjoyed the reputation of a special case (118), as categorization within taxonomies of emotions proved to be challenging and gave rise to controversies between scholars, especially in regards to whether it qualifies for the status of a primary emotion [for a review, see (119)]. Ekman & Friesen (120), for instance, disagreed with Izard’s (121) notion according to which contempt is to be subsumed under the response category of disgust, as they found contempt not only to be a distinct, but also a basic and therefore universal emotion. Krause (122) classifies contempt as a primary emotion with a distinct propositional structure and therefore delimits it from anger and disgust. In Affective Neuroscience (123), contempt has not clearly been taxonomically classified, especially because it did not seem to fit in one of the seven affective systems, found and described by Panksepp (29) using Deep Brain Stimulations (DBS) of subcortical brain regions. Tentatively, however, Montag & Panksepp (124) theorize contempt to arise from concurrent activity of the ANGER and the disgust circuitry. Additionally, they suggest contempt not to qualify as a primary emotion due to its lack of hot-headedness, which implies activity in the neocortex. However, inconsistent with this theory, contempt has been found to not only potentiate “cold” behaviors, but to also encompass a “hot” configuration, a so-called state of “boiling inward” (119, 125). Therefore, we concur with Ekman & Friesen (120) in classifying contempt as a primary emotion.

Despite the ongoing debates, there is a growing consensus regarding the nature of contempt. Contempt can be understood as the feeling that a person is beneath consideration (126). It is therefore always intentional and object-oriented (127), as well as associated with constant monitoring of another person’s value (128). Contempt is further understood to be the antithesis of respect (129), by way of which it is capable of muting prosociality (130) and even promoting terrorism (131). On a behavioral level, contempt is associated with a plethora of manifestations (119): largeness and downward glancing, disappointment signaling someone’s low value, as well as laughter and enjoyment, also coined as contemptuous delight (132). Additionally, contempt translates to outcomes of intolerance, exploitation, proactive social exclusion, derogation, rejection and relationship (119, 128).

More systematically, Gervais & Fessler (119) propose eight from the existing literature adduced features of which contempt consists: 1) it is intentional or about an object, 2) an enduring evaluation of a person, 3) it follows from cues to another’s low relation value, 4) entails loss of respect and status diminution, 5) creates “cold” indifference through diminished interest and muted prosocial emotions, 6) is associated with “anger” and “disgust”, 7) can be expressed in many ways, 8) leads to intolerance, exclusion and relationship dissolution.

Within a socio-functional approach to emotions (116, 133), contempt is conceptualized as consisting of three distinct but at the same time intertwined functions, two of which are particularly important for the present argumentation: (a) a self-regulation function and (b) a social distancing function (116). Self-regulatory and social-distancing aspects of contempt come to play whenever anger or hate is elicited, helping the contemptuous person to regulate themselves by devaluing the contempt-evoking object and subsequently distancing from it. Ultimately, contempt serves as a self-regulatory function by internally reducing the other to an extent that renders them worthless (117, 134). In this sense, contempt is also theorized to help boost feelings of self-worth and pride by viewing the other person as inferior and has also been theoretically linked to narcissism as a defensive affect against a wounded self (135).

Recently, dispositional contempt (i.e., contempt on the level of personality) was examined in regards to its nomological network, personality and behavioral correlates, as well as in regards to implications for relationship functioning (117). Not only was dispositional contempt found to be strongly associated with narcissism and envy, the study also found strong links between contempt and derogatory behaviors. Paradoxically, individuals high on dispositional contempt also reported strong feelings of loneliness and a need to belong – a finding that is particularly interesting when looked at through the lens of the narcissistic paradox outlined below (117).

Also, an important distinction is to be highlighted between the emotion of contempt and derogatory behaviors. While most of the existing models of narcissism accommodate derogation as a behavioral feature of antagonistic self-protection (8, 9, 87), they leave the emotional preconditions of these behaviors out of sight. By zooming in on the emotional substrate of derogatory behaviors, we aim to adopt a more nuanced approach at understanding what drives these behaviors, how they manifest exactly and in what interpersonal outcomes they result.

Following this argumentation and connecting theoretical and empirical accounts of contempt and envy with grandiose narcissism, while paying tribute to psychoanalytical theory, we posit that the emotion of contempt acts as an antidote against envy, just as Hotchkiss (136, p. 17) stated and Krizan & Johar (69) re-cited: “unaware of either envy or the need for superiority, these individuals may feel only self-righteous contempt”. Linking this further back to classic psychoanalytic theory, the result of contempt could be understood as so-called decathexis, a term that describes the process of withdrawing emotional energy from an object or a person (137). Building on this, we propose an emotion-centric self-regulatory process model of grandiose narcissism that integrates parts of the above-mentioned models and places this novel interplay of contempt and envy at the center of antagonistic self-protection.

## 8 Self-regulatory defensive affect model of grandiose narcissism

Our model seeks to integrate components of socio-functional approaches of emotion, Affective Neuroscience (21–23, 138, 139), as well as narcissism research (e.g. 2, 8, 9, 84, 87), in order to hypothesize an interplay between emotions which could shed light (a) on specific dynamics of rivalrous narcissism, (b) on how these dynamics explain interpersonal implications of narcissism and (c) on how social outcomes (i.e., conflict and potency) feed back into these dynamics.

In line with existing process models of narcissism, we understand the grandiose-narcissistic, especially antagonistic self-protective dynamic to be initiated whenever the overarching goal of status or grandiose-self maintenance is threatened (9, 140). Consistent with the SPIN-model (9), we understand narcissists to preferably select hierarchical situations, as they tend to prepare a fertile breeding ground for self-presentation. Additionally, we concur with the notion that grandiose narcissists are characterized by a heightened sensitivity (i.e., vigilance) to status-relevant cues in selected contexts (9, 87, 113). However, for narcissistic rivalry, we attribute both the situation selection and the vigilance aspects of the SPIN to dispositional envy, as the tendency to seek out hierarchical upwards comparisons as well as react sensitively on cues signaling undesirable comparison outcomes constitute inherent features of dispositional envy (93, 95, 141, 142).

**Figure 1:**
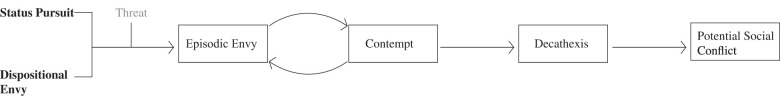
An overarching status motive coupled with dispositional envy catalyzes episodic envy when individuals high on grandiose narcissism are confronted with a status-threat. Contempt is elicited as a defensive emotion against envy, resulting in decathexis, which manifests itself as a lack of respect, indifference and derogation. This may ultimately lead to social conflict or relationship dissolution.

We further build on clinical-psychodynamic accounts (7, 12, 143) and Affective Neuroscientific theory (27), as well as socio-functional approaches of emotion (127, 133, 144–146) in order to hypothesize that self-protective narcissistic strategies aim to down-regulate envy once it is elicited by evoking contempt as a defensive affect. This chronic moderation of the relationship between grandiose narcissism and envy via contempt, we argue, constitutes the envy-protection suggested to be inherent to grandiose narcissism (69, 97). Far from being adaptive though, this dynamic has to be understood, in line with the EDER framework, as a dysregulated affective state that brings about other-derogative behaviors described in the NARC, as well as more specific contemptuous behaviors described above. Overall, we place this dynamic at the heart of grandiose- and more specifically narcissistic rivalry, concluding to the hypothesis that the antagonistic facet of narcissism stems from an emotional antagonism within. Ultimately, the elicitation of contempt may act as an antidote against the experience of envy, but is detrimental to social relationships at the same time – constituting what we call *envy’s Janus-faced antidote* (**Figure 1**).

Even though we integrate aspects of classic emotion-regulation approaches, such as the situation selection and the vigilance component that is also used in the SPIN, we decouple our model from a cognitive tradition in the strict sense by understanding (a) these cognitive functions to be inherent to emotions and (b) emotion regulation to result not so much from reappraisal but instead from the mobilization of defensive affects (33, 147–149). By conceiving of envy, contempt and the antagonistic interplay between the two emotions as the driving forces of rivalrous dynamics, our model also diverges from those that view emotions merely as outcome-variables [e.g (87).,]. That being said, the elicitation of contempt is not conceptualized as a conscious mental operation, let alone as a cognitive reappraisal strategy, but rather as an unconscious, automatic defense mechanism (147–150)

## 9 Interpersonal assumptions of the model

Social relationships are the playing field of intra- and interpersonal self-regulatory processes in general, as interpersonal maneuvers often serve intrapersonal needs and intrapersonal strategies often translate into interpersonal consequences (2, 9). To account for social relationships, especially close romantic relationships, our model also considers interpersonal dynamics and corresponding social outcomes, in line with most of the existing process models of narcissism (62, 151). We draw on findings from research on narcissism in relationship contexts that show agentic aspects of narcissism to be associated with short-term romantic appeal and narcissistic rivalry to be associated with social conflict in long-term acquaintances (2, 8, 60–62, 152–154). Similar to a chocolate cake (see *chocolate cake model*; 155), relationships with individuals high on grandiose narcissism are characterized by strong excitement at first, but by regret and conflict in later stages. These findings are usually explained by the change of priorities set by the partners in a more committed relationship: while at short-term acquaintance attributes such as attractiveness and charmingness are desirable, in later stages more communal attributes such as trustworthiness, warmth and altruism, are sought in a partner (61). These attributes, however, seem to not be compatible with narcissistic rivalry, as relationship research on narcissism has shown associations with a lack of interest and respect for their partners (61, 62, 156–160). However, the processes that underlie this change in relationship trajectory are still unclear. Can this phenomenon simply be attributed to a contextual change (i.e. short-term attraction of attractive- and charmingness vs. long-term attraction of warmth and trustworthiness) or to narcissistic behavior itself? Is relationship dysfunction present even at early relationship stages or does it emerge over time (161)? If so, do the positive effects of admiration wear off and give way to rivalrous behaviors that lead to conflict (61)?

We interpret previous data through the lens of psychodynamic accounts and our proposed framework. Given that our model understands rivalrous narcissistic dynamics to be initiated through an ego-threat that evokes envy, which in turn is defended against by an automatic elicitation of contempt, the question arises how this dynamic adds to the explanation of the relationship trajectories of narcissistic individuals. If, as stated repeatedly, individuals high on grandiose narcissism select situations, in which they think they can both self-present as well as upwardly compare themselves with others and if, in case of comparisons with negative outcome, they are confronted with envy, how can this be translated to close relationships? Put in other words: in what ways do individuals high on grandiose narcissism compare themselves to their relationship partners? To address this question, we come back to clinical accounts of envy. In psychoanalytic theory, envy is conceptualized as primarily addressed to social partners, however not necessarily because they are thought to have desirable possessions but instead because narcissists are confronted with strong feelings of dependency and powerlessness (7, 12, 143). In fact, the more a relationship advances, the stronger the mutual interdependence becomes (162). In order to defend against those feelings of dependency, powerlessness and inferiority, narcissists tend to disdain, devalue and, more importantly, feel contempt towards their social partners to an extent that leaves no room for positive views of them. Consistent with studies showing contempt as a key predictor of break-ups (163), we suggest that this dynamic leads to relationship dissolution. Further, we hypothesize that grandiose narcissists do not necessarily strive to regain their power, but rather to contemptuously devalue their social partners, depriving them of every desirable attribute they might have had, so that there is nothing left to feel dependent on, as well as nothing to be envious about. Put in other words: they unconsciously deprive themselves of the very relationships, that they so dearly longed for in their early childhood (7). This might suggest that the perception of dependency seems to create a special type of hierarchical upward comparison that evokes envy whenever the dependency is reactualized. In Morf & Rhodewalt’s model of narcissism (2), this has been connected to the construction of the narcissistic self and formulated as the ultimate narcissistic paradox: “as they yearn and reach for self-affirmation, they destroy the very relationships on which they are dependent.” (p. 179). Applied to the two strategies of grandiose narcissism, this could be understood as a process, in which the admiration pathway itself hinders its continuation (even though it boosts ego and status), as being dependent on the partner’s admiration is starting to be perceived as an ego-threat that needs to be defended against. Pointedly put: it may seem as though narcissistic rivalry infiltrates narcissistic admiration when feelings of dependency and power loss overwhelm individuals high on grandiose narcissism. This is also in line with the paradoxical findings that dispositional contempt is strongly linked to both narcissism as well as the need to belong (117). Drawing on this perspective, we hypothesize that close relationships present themselves as upward comparisons at some point and therefore entail an envy evoking potential which, in turn, elicits contempt.

Accordingly, we address the three above mentioned questions and suggest a novel explanation. Rather than attributing the change of relationship trajectory to either a contextual change or narcissistic behavior, in our model the observed relationship shift is conceptualized as an *interaction* between a contextual change together with a subsequent change in envy-evoking qualities of the context. In line with mentioned psychodynamic accounts, we hypothesize that as the relationship and emotions for the partners intensify, narcissists may start to feel dependent on either the affection or the admiration of their partner, perceiving the relationship in a way that resembles an upward comparison together with a loss of dominance or status. This, in combination with inevitably occurring micro-rejections within a relationship, may set in motion the proposed envy-contempt dynamic (and antagonistic dynamics in general), which ultimately leads to conflict (see below). In this vein, the potential for relationship dysfunction is present at early stages, but only exacerbates over time when envy emerges, while the positive effects of admiration wear off.

Preliminary evidence not so much for the specific hypothesis, but rather for status- and dominance-pursuit in close relationship contexts might support elements of the proposed theory. More broadly, antagonistic narcissism has been linked to the desire for power in close relationships (e.g., 164–167). More specifically, Wright et al. (168) found that perceptions of other’s dominance (i.e. the partner being “one-up”) predicted one’s own quarrelsomeness and that this effect was amplified by narcissism, suggesting this dynamic to contribute to the deterioration of relationships over time. Another recent study has revealed that the negative association between narcissistic rivalry and romantic relationship functioning was moderated by perceived power (169). Accordingly, the authors suggest that antagonistic behavior is spurred whenever individuals with higher levels of narcissistic rivalry are confronted with a loss of power in romantic relationships. Moreover, antagonistic narcissism has been found to be associated with status pursuit through the use of dominance-strategies, mediated by a competitive worldview (165). Furthermore, narcissistic admiration and rivalry has been investigated in terms of domain-specificity (170) and linked to social dominance as one of the playing fields in which self-promotional as well as other-derogative processes are acted out. These findings may act as supporting evidence for the hypothesis that a loss of power may trigger status pursuit through dominance-based strategies and this dynamic to be characteristic for antagonistic narcissism. However, whether loss of power or dominance is associated with increased dependency on the social partner’s admiration remains to be examined.

## 10 Social conflict

In agreement with existing models and correspondent findings, we understand antagonistic narcissism to result in social conflict caused by aggressive behaviors, which can be defined as any form of behavior directed toward the goal of harming another person who is motivated to avoid such treatment (83)However, our understanding of social conflict and the way it feeds back into the rivalrous dynamic somewhat differs from other models. While in most models, social conflict is understood as an ego-threat itself and therefore to feed back into and subsequently reinstate the antagonistic self-protection (8), we hypothesize that once contempt and the derived behavioral consequences, such as derogation and devaluation, culminate, the social partner is no longer perceived as worthy enough to act either as a source of status or as a threat to it. As outlined above, contempt seeks to render the social partner worthless, in order to facilitate both distancing from them as well as feeling indifferent towards their judgement.

We therefore understand social conflict to emerge from a more complex emotional process (171, 172), that is spurred by the proposed envy-contempt dynamic, which in turn does not necessarily directly lead to aggressive transgressions, but more likely to indifference, irritation and annoyance – characteristic behaviors of contemptuous persons and possibly precursors of aggressive behavior. In line with Gervais & Fessler (119), we argue that contempt disposes aggressions towards the contemned, but does not intrinsically motivate harming that person. This might preliminary be in line with previous studies that examined the link between narcissism and aggression. Back et al. (8) found links between antagonistic narcissism and aggressive behaviors, however only assessed mild aggression or internal precursors of aggression (i.e. annoyance or irritation) (8). Krizan & Johar (11) did not find grandiose narcissism to amplify aggressive behavior in response to interpersonal frustration. Moreover, associations between perception of low status success and hostile behaviors received less consistent support, leading to the conclusion that loss of status does not necessarily lead to overt aggression but may as well trigger flight, rather than fight responses (173, 174). On the other hand, there are multiple meta-analyses that robustly connect all forms of narcissism (i.e., grandiose and vulnerable to all forms of aggression (i.e., indirect, direct, physical) (83, 175).

In short, while findings strongly support the link between narcissism and aggression, the multifaceted nature of the underlying processes however is somewhat unclear. Contempt as a main contributor to the emergence of aggression and potentially as a moderator of the link between narcissism and aggression has been examined in a few studies thus far, providing useful hints to our proposed hypothesis. Dispositional contempt (i.e. contempt as a trait) has been found to be strongly linked to narcissism (117) and to moderate the relationship between psychopathy, which is one of the four personality traits of the Dark Tetrad and therefore closely related to narcissism, and both reactive and proactive aggression (176).

Taken together, while the interactional outcome of antagonistic self-protection may well be social conflict, we interpret the given data not so much as open aggression and social conflict, but more as a possible precursor of the latter. What does this mean for the way the antagonistic self-protective dynamic perpetuates itself? In contrast to models that understand social potency to feed back into the motivational dynamic of narcissism in the form of an ego-boost and social conflict in the form of an ego-threat we suggest that when contemptuous devaluation culminates, the social partner is perceived as no longer worthy enough to provide an ego-boost nor an ego-threat. Subsequently, the narcissistic individual is likely to socially distance themselves, while at the same time looking out for potential new partners. Accordingly, while we hypothesize the rivalrous narcissistic dynamic not to feed back in form of ego-threat, we suggest that it undermines the possibility of garnering respect or admiration, not so much because the partner is not willing to provide it, but rather because their admiration is not perceived as important enough to boost ego any longer.

## 11 Future research and validation

Existing direct and indirect empirical evidence for aspects of the model has been laid out over the course of this article. Generally, however, the presented model is of theoretical nature and naturally lacks empirical scrutiny. Therefore, future research is needed to examine and validate the proposed processes.

Although both dispositional and episodic contempt have started to garner scientific interest, only the few studies presented above have examined the link between dark-triad personality traits and dispositional contempt (117, 176). These studies, however, make use of unitary constructs of narcissism, which usually fail to capture the complexity of the phenomenon. In future research, we suggest the NARC to be used in order to examine links between narcissistic rivalry and contempt. Furthermore, and in line with suggestions formulated by Tracy et al. (84), regarding the measurability of pride and shame through non-verbal behaviors, a similar suggestion can be made in regards to contempt. As outlined above, the emotion of contempt is controversial but nevertheless mostly conceptualized as a distinct basic emotion that is expressed invariantly and universally (120, 177, 178). Accordingly, either the *Facial Action Coding System* (FACS; 179) or according software built to automatically analyze facial expressions could be used in order to measure contempt *in vivo*. Moreover, physiological measures, such as facial electromyography, could be used to further eliminate disadvantages of self-reports, as has been done in a recent study (86).

In terms of associations between narcissism and envy, there is a growing but yet elusive body of evidence, with a number of studies suggesting grandiose narcissists to be immune to envy (69, 97), while other studies substantiate links between narcissistic rivalry and malicious forms of envy (93). However, even though the latter found robust positive links between narcissistic rivalry and malicious envy and therefore might be thought to weaken our argumentation (according to which grandiose narcissism curbs envy feelings stably), zooming in on the used measures is insightful and might even be interpreted in favor of our hypothesis. For example, items used to measure malicious envy include such as “feeling cold towards a person” and “having negative thoughts about a person” (93), both items that are reminiscent of contempt features. Particularly “feeling cold” has been included in the only scale measuring dispositional contempt, the Dispositional Contempt Scale (DCS; 117). Consequently, we argue that the link between the two facets of grandiose narcissism and envy remains elusive, with a strong indication that grandiose narcissism protects against envy. Because our model is grounded on this particular repression theory of envy, it should be further examined in order for the empirical groundwork to be solidified, preferably under experimental conditions that allow for social comparisons or status-threats to be manipulated. We use the term repression in line with Horvath & Morf (113), who point out that repression is instigated by an unconscious intention to keep unwanted emotions and cognitions out of awareness and runs in two consecutive stages, namely vigilance and avoidance. Repression can be used both adaptively and maladaptively, depending on how rigidly or constructively it is used. We further add that contempt as a defensive affect can be attributed to the avoidance stage of repression.

This leads to our main hypothesis: the antagonistic interplay between envy and contempt, which we hypothesize to be constitutive for antagonistic self-protection. While this hypothesis has received considerable attention in psychodynamic literature and is a widely held clinical assumption (7, 180), it has not yet been subject of systematic empirical scrutiny. Indeed, empirically examining this hypothesis is a somewhat challenging endeavor, as conceptualizing envy as existent but at the same time repressed may be reminiscent of the classic concept of the unconscious, which per definition is hardly accessible to direct assessment (181). However, this is not applicable to our case, as we propose antagonistic self-protection to curb envy feelings, which can simply be understood as a negative correlation between the two constructs, consistent with supporting evidence. We suggest the negative relationship between grandiose narcissism and envy to be amplified by the moderation via contempt, which is why future moderation analyses may seem particularly purposeful. Specifically, we assume contempt to act as a suppressor variable, weakening the effect between grandiose narcissism and dispositional envy by its omission (182, 183). This may present a potential explanation for the negative, but nonsignificant predictive validity of grandiose narcissism for dispositional envy.

Furthermore, we believe experimental designs to be best suited for validating this hypothesis, as we conceive of the envy-contempt dynamic to be set in motion through the perception of a status-threat. Prior studies have used staged social exclusions (184, 185) or rigged computer games (140) to induce status-threats. An ideal design would combine self-reports for narcissism, both the PNI to assess grandiose and vulnerable dimension, as well as the NARC to zoom in on narcissistic rivalry and admiration, self-reports for envy and contempt and non-verbal methods, such as the FACS or software based equivalent, that are able to monitor facial action related to contempt while the status-threat is presented.

In regards to interpersonal implications of our model, longitudinal designs seem to be best suited for assessing empirical validity. In line with empirical evidence (61), our model conceives of assertive self-enhancement to contribute to relationship success at short-term acquaintance and, on the contrary, antagonistic self-protection to unfold in long-term relationships, giving rise to romantic problems. We draw on psychodynamic accounts to add to the understanding of the underlying processes of this trajectory change. Specifically, we assume that individuals high on grandiose narcissism turn against the persons that they receive positive feedback from, simply because they begin to feel like they depend on their admiration, resulting in a perceived loss of power and dominance. Indeed, social dominance has been found to be one of the playing fields, on which both self-enhancement as well as self-protective strategies are acted out (170). The perception of loss of social dominance subsequently catalyzes antagonistic self-protection, ultimately resulting in relationship conflicts through the proposed envy-contempt dynamic. To test this turning-against-the-admirer hypothesis, longitudinal studies on how grandiose narcissists perceive their level of social dominance in their close relationships over time seem purposeful.

Lastly, further empirical scrutiny is needed in regards to the outcomes and feedback dynamics our model proposes. As outlined above, our conceptualization of social conflict and the way it feeds back into the cycle of status-pursuit diverges from existing models. The model suggests that contemptuous devaluation leads to a loss of interest and respect, as the partner is no longer perceived as worthy enough to interact with (116, 119). The resulting emotional dis-investment may well prepare a fertile breeding ground for social conflict, however is not equal to it. Furthermore, according to our model, antagonistic self-protection paves the way for relationship dissolution via contempt (119) but does not perpetuate itself through feeding back into the rivalrous dynamic, setting the process in motion anew. Instead, it subverts any possibility of reconciliation, as the social partner successively loses their worth and is therefore incapable of providing admiration. Because this hypothesis is derived from outcomes associated with contempt, future research in regards to grandiose narcissism and (dispositional) contempt should be helpful in establishing these links. Moreover, as already outlined above, meta-analyses have found all forms of narcissism to be strong predictors of all forms of aggression (83). Also, consistent with the findings that dispositional contempt strengthens the link between dark-triad traits and aggression (176), similar results could be expected in terms of narcissistic rivalry and aggression. Accordingly, we suggest future research to examine whether both dispositional and episodic contempt amplify the link between narcissistic rivalry and both reactive as well as proactive aggression.

## 12 Similarities and differences to existing models

Our model has manifold similarities to existing models. In line with the NARC (8) and the SPIN (9), it places status-pursuit as the overarching goal of grandiose narcissism. Moreover, it understands antagonistic self-protection to be set in motion through the perception of an ego- or status-threat. Emotions, such as envy and anger (i.e., 87), are emphasized, designing the model as an emotion-centered approach (i.e., 84). Also, social conflict as the social outcome of antagonistic self-protection is stressed.

However, our model also differs from existing models in many ways. On a conceptual level, it combines theory and empirical findings from a plethora of disciplines: classic and contemporary psychoanalytic theory (7, 12), Affective Neuroscience (21–23, 27, 33, 138, 139), and socio-functional approaches of emotions (116, 133). In line with this conceptual framework but inconsistent with existing models of narcissism, emotions are placed at the center of antagonistic self-protection, rather than reduced to emotional outcomes of cognitive appraisals. Furthermore, we focus on specific emotions, envy and contempt – two emotions that are generally underrepresented in existing models of narcissism (with two exceptions regarding envy, see 87, 93). Additionally, in line with the outlined EDER framework, these emotions are understood to antagonize with each other, constituting a dysregulated affective state due to defensive affects. As alluded to above, this defense-oriented approach is inspired by clinical accounts and object-relations theory and therefore contributes to contemporary reformulations of classic psychoanalytic theory (e.g. (186–188).

Our model therefore transcends the limits of emotion regulation concepts that rely on appraisal theory and cognitive tradition, by building on findings and theory from Affective Neuroscience (24). This allows us to conceptualize emotions and their implicit regulation as the main driving forces of personality processes, while at the same time granting psychodynamic accounts entry into our model. The strengths but also the limitations of our model are presented in the table below (**Table 1**).

**Table 1:** Strengths and Limitations of the Model.

Category	Strengths	Limitations
Conceptual	- Integrates recent advances and findings from several disciplines - Is informed by contemporary neuroscience - Translates psychodynamic theories into testable hypotheses	- Places limited emphasis on cognitive processes - Potentially overemphasizes psychodynamic theory and its contemporary reformulations - Is only partially supported by empirical evidence
Explanatory	- Explains narcissistic behaviors as a result of two specific emotions - Understands emotions not just as outcomes but as driving forces behind narcissistic traits - Deepens the understanding of derogatory behaviors that are characteristic of narcissism - Provides a compelling explanation for the envy-resistance typical of grandiose narcissism - Addresses open questions in research on relationship functioning of individuals high in grandiose narcissism - Enhances understanding of precursors to social conflict	- Exclusive focus on envy and contempt may be reductionist - The conceptual distinction between contempt and devaluation may be insufficiently defined - Focuses only on antagonistic self-protection, excluding other forms of narcissism

## 13 Clinical and societal significance

Shedding light on these dynamics is not only of scientific relevance but also carries significant clinical and societal implications. From a clinical standpoint, it is as crucial as it is challenging to recognize indifference and a lack of interest as the expression of a highly complex process. More often than not, individuals suffering from pathological narcissism tend to disdain and devalue their therapist—paradoxically at the very moment they begin to perceive the therapist as a helpful and caring figure. This dynamic, originally termed by Klein (12) as an envy-based form of the negative therapeutic reaction and later validated by contemporary object-relational therapists (7), can be deeply detrimental to therapy. It can only be fully understood when considering that feelings of dependency may evoke envy, which is then defensively countered through the emergence of contempt, precisely as outlined in our model.

On a societal level, our model may not only help individuals high in grandiose narcissism to better reflect on their thoughts, feelings, and behaviors within romantic relationships, but also support their partners through psychoeducation—enabling them to navigate these relationships more effectively and ultimately cope with relationship dissolution.

## 14 Conclusion

In this article, we combined empirical evidence and theory from psychodynamic accounts, Affective Neuroscience, socio-functional approaches of emotion as well as social- and personality psychology to propose a framework that aims to shed light on emotional processes of grandiose narcissism. By interpreting given findings through the lens of our model, we hypothesize that individuals high on narcissistic rivalry react to status threat with strong feelings of envy that are down-regulated through the automatic elicitation of contempt. We further hypothesize that contempt translates into derogatory and devaluing behaviors characteristic to narcissism in general and in lack of interest, indifference, exploitation, intolerance, exclusion and contemptuous delight more specifically. Moreover, we conceive of these behaviors to be precursors of aggressions. Ultimately, we believe that contempt gradually subverts social relationships by rendering relationship partners worthless, inevitably leading to relationship dissolution.

## Data Availability

The original contributions presented in the study are included in the article/supplementary material. Further inquiries can be directed to the corresponding author.
